# Aporé virus, a novel mammarenavirus (Bunyavirales: Arenaviridae) related to highly pathogenic virus from South America

**DOI:** 10.1590/0074-02760180586

**Published:** 2019-05-23

**Authors:** Jorlan Fernandes, Alexandro Guterres, Renata Carvalho de Oliveira, Rodrigo Jardim, Alberto Martín Rivera Dávila, Roger Hewson, Elba Regina Sampaio de Lemos

**Affiliations:** 1 Fundação Oswaldo Cruz-Fiocruz, Instituto Oswaldo Cruz, Laboratório de Hantaviroses e Rickettsioses, Rio de Janeiro, RJ, Brasil; 2 Fundação Oswaldo Cruz-Fiocruz, Instituto Oswaldo Cruz, Laboratório de Biologia Computacional e Sistemas, Rio de Janeiro, RJ, Brasil; 3 Public Health England, National Infection Service, Salisbury, United Kingdom

**Keywords:** arenavirus, mammarenavirus, *Oligoryzomys mattogrossae* rodent, Aporé virus

## Abstract

Here, we report the complete genome sequence of the Aporé virus (*Bunyavirales*: *Arenaviridae*), obtained from a wild rodent *Oligoryzomys mattogrossae* captured in Mato Grosso do Sul state, Brazil. The genome of this virus showed strong similarity to highly pathogenic mammarenavirus from South America.

Mammarenaviruses are negative sense bi-segmented RNA viruses each segment encodes genes for two non-overlapping reading frames in ambisense polarity.^1^ The *Arenaviridae* family currently comprises 41 viral species, classified into three genera: *mammarenavirus* (35 species), *reptarenavirus* (five species), and *hartmanivirus* (one specie).^2^ Most mammarenaviruses have natural reservoirs in rodents and are historically classified into two groups according to their genomic features and antigenic properties: the Old World Lassa-Lymphocytic choriomeningitis virus (LCMV) serocomplex, including viruses from Africa and, recently, Asia; and the New World Tacaribe serocomplex, formed by viruses indigenous to the Americas and segregated into clade A, B, C and D (clade D was formerly known as Clade A recombinant).^1^ ^,^ ^2^

To date, eight mammarenavirus were detected in Brazil: Amaparí virus (*Neacomys guianae*), Cupixi virus (*Oryzomys megacephalus*), Flexal virus (unidentified oryzomyini rodent), Sabiá virus (unknown animal reservoir), Oliveros virus (*Necromys lasiurus*), Latino virus (*Calomys callosus* and *C. callidus*), Pinhal virus (*Calomys tener*) and Xapuri virus (*Neacomys musseri*).^1^ ^,^ ^2^ ^,^ ^3^ ^,^ ^4^ Here, we report the complete genome characterisation of a novel mammarenavirus detected in field collected specimens of *Oligoryzomys mattogrossae* (= *Oligoryzomys fornesi*), captured in Cassilândia municipality, Mato Grosso do Sul state, Midwest, Brazil.^5^

Whole genome sequencing was performed using Illumina HiSeq 2500 sequencer (Illumina Inc, USA). Isolated RNA from one rodent liver sample was treated with DNAse I (Life Technologies) following the manufacturer’s instructions and depleted of ribosomal RNA using NEBNext rRNA Depletion Kit (New England BioLabs inc). A library was constructed with the Nextera XT Library Preparation Kit (Illumina) using 2 x 250 bp paired-end protocol on the MiSeq platform (Illumina). Sequencing reads were assembled *de novo* using CAP3 and MIRA 3.9.18 performed using a local instance of *Stingray@Galaxy* based on the Galaxy Project.^6^ ^,^ ^7^ Coding for complete sequences of both segments were loaded into the Pairwise Sequence Comparison (PASC) tool, and analysed using the default parameters.^8^

A classical arenavirus bi-segmented genome was identified, each segment encoding two open reading frames (ORFs) in an ambisense organisation with an intergenic region containing a predicted stem-loop region between the ORFs. Full S segment (3.4 kb) encoded genes for two inferred proteins: nucleoprotein (562 aa) and a glycoprotein precursor (GPC) (489 aa), which is normally post-translationally processed into the envelope glycoproteins G1 (197 aa) and G2 (234 aa) and the stable signal peptide (SSP - 58 aa). The L segment (7.2 kb) encoded genes for zinc-binding matrix protein (99 aa) and the RNA-dependent RNA polymerase (2155 aa). Additional features commonly observed in mammarenavirus genomes include the conservation of the 3′-5′ termini and the presence of an L-domain motif within the Z protein.

Nucleotide sequences and deduced amino acid of the new virus were compared to those of other mammarenaviruses species. A nucleotide sequence divergence of 28% for S and 25% and 26% for L segment was found between the new virus and Chapare (from Bolivia) and Sabiá (from Brazil) viruses, respectively.^9^ ^,^ ^10^ Interestingly the new virus and Chapare viruses (CHAPV) were also closely related at their structural proteins: RNA-dependent RNA polymerase (74%), zinc-binding matrix (66%) and nucleoprotein (87%). Curiously, the glycoprotein precursor was slightly more related to SABV, with 81% of identity, indicating that recombination events have played a significant role in its evolution.

Pairwise sequence comparison (PASC) was performed on both segments reinforcing the close relation between the new discovered virus from *O. mattogrossae*, SABV and CHAPV. Thus, the results of the phylogenetic analysis also indicates that it represents a novel virus within the Clade B New World *mammarenavirus* (*Bunyavirales*: *Arenaviridae*) (Figure). Therefore, we suggest naming it Aporé mammarenavirus, after a river close to the site where the rodent specimens were collected, with the abbreviation APOV.

In recent years, novel arenaviruses have been identified expanding our knowledge about their genetic diversity, geographic range and host association. Herein we show that APOV is closely related to two highly pathogenic arenaviruses from South America that were recovered from fatal cases of hemorrhagic fever, whose reservoirs remain unknown.^9^ ^,^ ^10^ More studies are needed to elucidate the epizootiologic aspects of this novel mammarenaviruses, in order to better understand the dynamics involving *O. mattogrossae* rodents and APOV.

**Figure:**
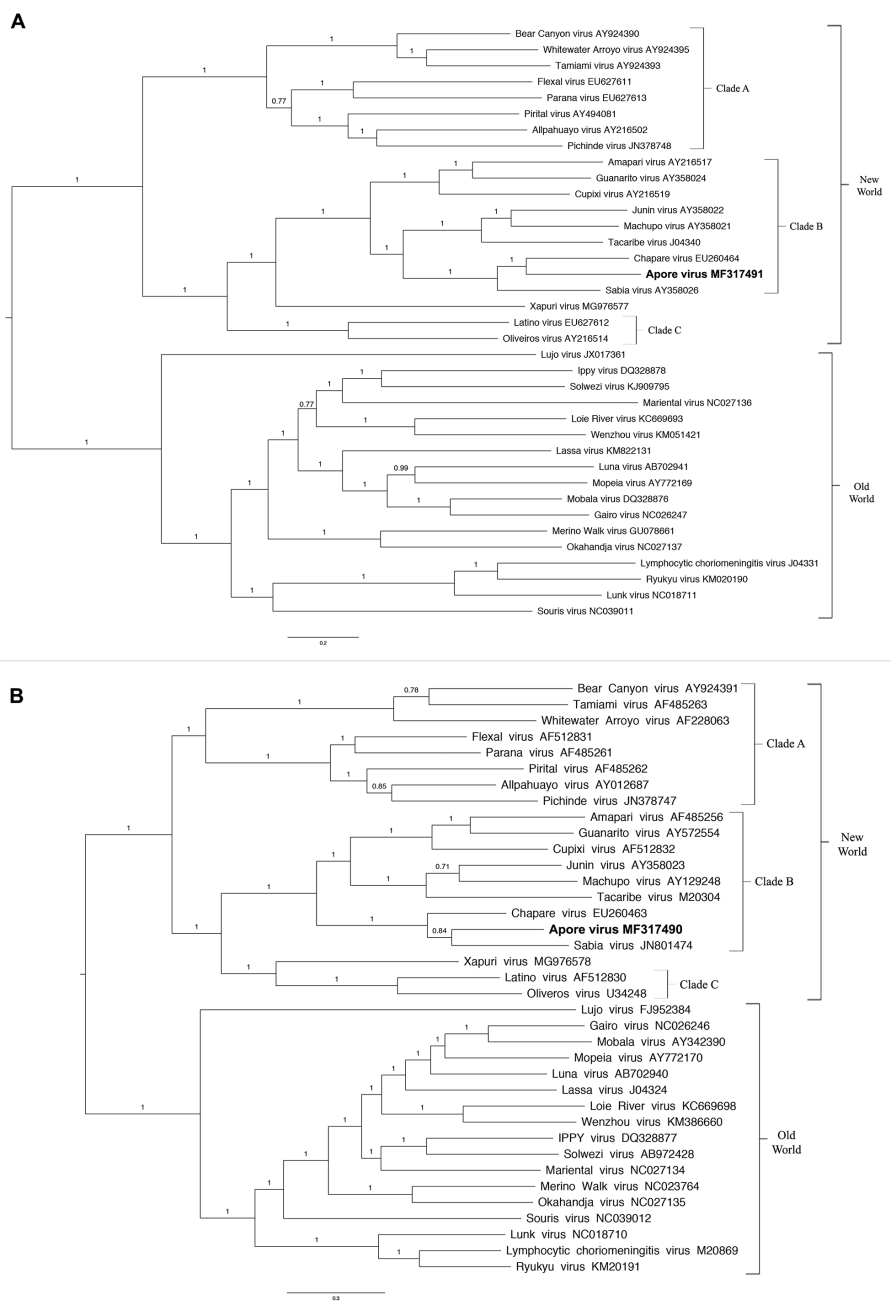
Phylogenetic tree based on mammarenaviruses complete L (A) and S (B) segments, Bayesian method (MrBayes v3.2.5.), using the evolutionary model GTR+G+I. Numbers (≥ 0.7) above branches indicate posterior node probabilities. Sequences of this study are highlighted in bold.

*Nucleotide sequence accession numbers* - The complete genome sequence of Aporé virus has been deposited in GenBank under the following accession numbers: MF317490 for the S segment, and MF317491 for the L segment.

## References

[1] Radoshitzky SR, Bào Y, Buchmeier MJ, Charrel RN, Clawson AN, Clegg CS (2015). Past, present, and future of arenavirus taxonomy. Arch Virol.

[2] Maes P, Alkhovsky SV, Bào Y, Beer M, Birkhead M, Briese T (2018). Taxonomy of the family Arenaviridae and the order Bunyavirales update 2018. Arch Virol.

[3] Fernandes J, Oliveira RC, Guterres A, Barreto-Vieira DF, Terças ACP, Teixeira BR (2018). Detection of Latino virus (Arenaviridae Mammarenavirus) naturally infecting Calomys callidus. Acta Trop.

[4] Fernandes J, Guterres A, de Oliveira RC, Chamberlain J, Lewandowski K, Teixeira BR (2018). Xapuri virus, a novel mammarenavirus natural reassortment and increased diversity between New World viruses. Emerg Microbes Infect.

[5] Weksler M, Lemos EMS, D'Andrea PS.Bonvicino CR (2017). The taxonomic status of Oligoryzomys mattogrossae (Allen 1916) (Rodentia Cricetidae: Sigmodontinae), Reservoir of Anajatuba hantavirus. Am Mus Novit.

[6] Wagner G, Jardim R, Tschoeke DA, Loureiro DR, Ocaña KA, Ribeiro AC (2014). STINGRAY system for integrated genomic resources and analysis. BMC Res Notes.

[7] Huang X, Madan A (1999). CAP3 a DNA sequence assembly program. Genome Res.

[8] Bao Y, Chetvernin V, Tatusova T (2014). Improvements to pairwise sequence comparison (PASC) a genome-based web tool for virus classification. Arch Virol.

[9] Delgado S, Erickson BR, Agudo R, Blair PJ, Vallejo E, Albariño CG (2008). Chapare virus, a newly discovered arenavirus isolated from a fatal hemorrhagic fever case in Bolivia. PLoS Pathog.

[10] Coimbra MTL, Nassar ES, Burattini MN, de Souza LT, Ferreira I, Rocco IM (1994). New arenavirus isolated in Brazil. Lancet.

